# Prescribing of Antidiabetic Medicines before, during and after Pregnancy: A Study in Seven European Regions

**DOI:** 10.1371/journal.pone.0155737

**Published:** 2016-05-18

**Authors:** Rachel A. Charlton, Kari Klungsøyr, Amanda J. Neville, Sue Jordan, Anna Pierini, Lolkje T. W. de Jong-van den Berg, H. Jens Bos, Aurora Puccini, Anders Engeland, Rosa Gini, Gareth Davies, Daniel Thayer, Anne V. Hansen, Margery Morgan, Hao Wang, Anita McGrogan, Anne-Marie Nybo Andersen, Helen Dolk, Ester Garne

**Affiliations:** 1 Department of Pharmacy and Pharmacology, University of Bath, Bath, United Kingdom; 2 Medical Birth Registry, The Norwegian Institute of Public Health, Bergen, Norway; 3 Department of Global Public Health and Primary Care, University of Bergen, Bergen, Norway; 4 IMER (Emilia Romagna Registry of Birth Defects), Center for Clinical and Epidemiological Research, University of Ferrara, Ferrara, Italy; 5 Department of Nursing, College of Human and Health Sciences, Swansea University, Swansea, Wales, United Kingdom; 6 Institute of Clinical Physiology—National Research Council (IFC-CNR), Pisa, Italy; 7 Pharmacoepidemiology and Pharmacoeconomics unit, Department of Pharmacy, University of Groningen, Groningen, The Netherlands; 8 Drug Policy Service, Emilia Romagna Region Health Authority, Bologna, Italy; 9 Department of Pharmacoepidemiology, The Norwegian Institute of Public Health, Bergen, Norway; 10 Agenzia Regionale di Sanità della Toscana, Florence, Italy; 11 Centre for Health Information, Research and Evaluation, Swansea University, Swansea, Wales, United Kingdom; 12 Paediatric department, Hospital Lillebaelt, Kolding, Denmark; 13 CARIS, The Congenital Anomaly Register for Wales, Singleton Hospital, Swansea, United Kingdom; 14 Section of Social Medicine, Department of Public Health, University of Copenhagen, Copenhagen, Denmark; 15 Centre for Maternal, Fetal and Infant Research, Institute for Nursing and Health Research, Ulster University, Newtownabbey, Northern Ireland, United Kingdom; Medical Clinic, University Hospital Tuebingen, GERMANY

## Abstract

**Aim:**

To explore antidiabetic medicine prescribing to women before, during and after pregnancy in different regions of Europe.

**Methods:**

A common protocol was implemented across seven databases in Denmark, Norway, The Netherlands, Italy (Emilia Romagna/Tuscany), Wales and the rest of the UK. Women with a pregnancy starting and ending between 2004 and 2010, (Denmark, 2004–2009; Norway, 2005–2010; Emilia Romagna, 2008–2010), which ended in a live or stillbirth, were identified. Prescriptions for antidiabetic medicines issued (UK) or dispensed (non-UK) during pregnancy and/or the year before or year after pregnancy were identified. Prescribing patterns were compared across databases and over calendar time.

**Results:**

1,082,673 live/stillbirths were identified. Pregestational insulin prescribing during the year before pregnancy ranged from 0.27% (CI_95_ 0.25–0.30) in Tuscany to 0.45% (CI_95_ 0.43–0.47) in Norway, and increased between 2004 and 2009 in all countries. During pregnancy, insulin prescribing peaked during the third trimester and increased over time; third trimester prescribing was highest in Tuscany (2.2%) and lowest in Denmark (0.5%). Of those prescribed an insulin during pregnancy, between 50.5% in Denmark and 88.8% in the Netherlands received an insulin analogue alone or in combination with human insulin, this proportion increasing over time. Oral products were mainly metformin and prescribing was highest in the 3 months before pregnancy. Metformin use during pregnancy increased in some countries.

**Conclusion:**

Pregestational diabetes is increasing in many areas of Europe. There is considerable variation between and within countries in the choice of medication for treating pregestational diabetes in pregnancy, including choice of insulin analogues and oral antidiabetics, and very large variation in the treatment of gestational diabetes despite international guidelines.

## Introduction

The prevalence of pregestational diabetes has been increasing in women of child-bearing age in recent years,[1] partly as a result of changes in diet and lifestyle which is associated with type 2 diabetes. Diabetes, and particularly poorly controlled diabetes, is associated with an increased risk of pregnancy complications and adverse pregnancy outcomes for both the mother and the fetus/child.[2–9] The St Vincent Declaration of 1989 advocated that within five years women with diabetes should have approximately the same proportion of adverse pregnancy outcomes as women without diabetes.[10] This has yet to be achieved.[11] National guidelines recommend that women with diabetes who are planning to become pregnant attend pre-conception counselling and aim for good glycaemic control, in order to reduce the risk of complications and adverse pregnancy outcomes.[12]

In addition to women with pre-existing type 1 and type 2 diabetes, between 2% and 10% of pregnancies in Europe are affected by gestational diabetes.[13] Screening for gestational diabetes is therefore recommended in national guidelines for women considered to be at an increased risk.[12, 14] The threshold for diagnosis of gestational diabetes has been lowered in some countries[15] leading to increasing numbers of diagnosed cases, but the increase is also due to changes in lifestyle and screening.[13, 16]

For some patients with type 2 or gestational diabetes glycaemic control can be improved through changes in diet and exercise. For others, however, treatment with either insulin or oral antidiabetic medicines is required. The traditional treatment for type 1 diabetes and some type 2 and gestational diabetes has been with human insulin. Since the mid-1990s, however, insulin analogues have become available and these are synthetic insulins with better pharmacokinetic profiles than human analogues. The rapid-acting insulin analogues have the advantage of a more rapid onset of action than human insulin and the long-acting analogues are released more slowly, without a peak in activity, providing more stable blood levels for a longer duration.[9] There has been debate about the safety of oral antidiabetics such as metformin in pregnancy, but recent UK guidelines[17] and a meta-analysis[18] suggest that there is now evidence relating to the efficacy and safety of metformin use for the treatment of gestational diabetes. This study aimed to describe and compare the prescribing of different antidiabetic medicines before, during and after pregnancy in seven regions of Europe.

## Methods

Seven population-based electronic healthcare databases, that captured pregnancies and prescription data, were accessed: two in Italy (Tuscany[19] and Emilia Romagna[20]), two in the United Kingdom (the Secure Anonymised Information Linkage [SAIL] Databank in Wales[21, 22] and the UK-wide Clinical Practice Research Datalink [CPRD][23] with data from Wales excluded) and one in Denmark,[24–26] Norway[27, 28] and the Netherlands.[29] A more detailed description of the databases can be found elsewhere [30] and an overview of the data available within each of the different databases and how they are collected is provided in S1 Table.

A common protocol and data specification were used to ensure that the data extracted from each database were as comparable as possible. All live and stillbirths born at ≥20 weeks gestational age, where the pregnancy started and ended between 1 January 2004 and 31 December 2010, were identified in the seven databases (in Denmark, Norway and Emilia Romagna, owing to data availability, the inclusion dates were 1-Jan-2004 to 31-Dec-2009, 1-Jan-2005 to 31-Dec-2010 and 1-Jul-2008 to 31-Dec-2010 respectively). Pregnancies were excluded if the mother had not been followed in the database which captured prescription data for the entire pregnancy and for the year before and after pregnancy. For each eligible pregnancy, the start date of the pregnancy was extracted or estimated based on additional data such as gestational age at delivery (S1 Table). For each pregnancy, the start and end of each pregnancy trimester was determined; trimester one was from the start of pregnancy through to 90 days gestational age, trimester two was from 91 until 188 days and trimester three was from 189 days gestational age until delivery.

All prescriptions for an antidiabetic medicine recorded in the databases during the time period of interest were identified. In the UK/Wales this included all prescriptions issued in primary care whilst in other regions it included all prescriptions dispensed by a pharmacist. None of the databases captured medicines given directly to the patient during a hospital stay. In the Netherlands, Denmark and Norway the dispensing of all other antidiabetic medicines was captured. In the UK databases, prescriptions issued by a specialist in a hospital outpatient department and private prescriptions were rarely recorded; these numbers were likely to be small as most subsequent repeat prescribing will have been undertaken in primary care and private practice is limited. In Italy, only prescriptions reimbursed by the Italian healthcare system were captured; this excluded private prescriptions. In Emilia Romagna, prescriptions directly dispensed at a hospital pharmacy were not captured until 2008 and this accounted for over 75% of insulin prescriptions (A Puccini—personal correspondence). In Tuscany, however, prescriptions dispensed at hospital pharmacies were captured throughout the study period.

Insulins investigated were human, analogue and pre-mixed combinations categorised as having an Anatomical Therapeutic Chemical (ATC) classification code starting with A10A. Oral antidiabetic products were those with an ATC code starting with A10B and included, but were not limited to, biguanides, sulfonylureas, sulfonamides, combinations of oral blood glucose lowering drugs and thiazolidinediones.

Data on the indication for prescribing was not evaluated as part of this study as this information was not commonly recorded in many of the databases. For insulins, the indication will always have been diabetes but metformin is also prescribed in fertility clinics to help women conceive and for treatment of polycystic ovary syndrome.

### Analyses

The percentage of women receiving a prescription for insulin or an oral antidiabetic medicine, in each of the databases, was calculated for the year before pregnancy, during pregnancy and the year following pregnancy. The prevalence of prescribing was also described for each pregnancy trimester and for 3-month time periods during the years before and after pregnancy. Women who had two pregnancies in close succession, where part of the 1-year time period following a pregnancy overlapped to some extent with part of the 1-year time period before the start of a subsequent pregnancy, were eligible for inclusion in both the pre-pregnancy and post-pregnancy analyses. The prevalence of prescribing of specific products and also the proportion of women prescribed human insulin, insulin analogues or a combination of the two was evaluated for each database. Prescribing of oral antidiabetic medicines was also evaluated, along with changes in prescribing patterns during the study period. To evaluate the impact of differences in maternal age between regions, the prevalence of prescribing of insulins during each of the 3-month time periods was calculated, for each region, age-standardised to the Eurostat birth figures for 2007.[31]

In each database, the percentage of deliveries where the woman had no prescription for an antidiabetic medicine during the year before pregnancy or the first trimester but received her first prescription during the second or third trimester of pregnancy, was calculated to provide an estimate of those affected by gestational diabetes who received pharmacological treatment.

## Results

### Prevalence of antidiabetic medicine prescribing

A total of 1,082,673 deliveries were identified within the seven databases and for 21,669 (2.0%) the mother received a prescription for an antidiabetic medicine in the year before, during or the year following pregnancy. The mean maternal age at the start of pregnancy for the entire cohort ranged from 27.6 years in Wales to 32.2 years in Emilia Romagna (Table 1).

**Table 1:** Percentage of deliveries where the woman received a prescription for insulin or an antidiabetic medicine in the year before pregnancy, during pregnancy or the year following pregnancy.

Country/region	Entire study cohort		The woman received ≥1 prescription for insulin						The woman received ≥1 prescription for anoral antidiabetic medicine					
	Number of deliveries	Mean maternal age^a^	Year before pregnancy		During pregnancy		Year after pregnancy		Year before pregnancy		During pregnancy		Year after pregnancy	
	N	Years [SD^b^]	(%)	(95% CI)	(%)	(95% CI)	(%)	(95% CI)	(%)	(95% CI)	(%)	(95% CI)	(%)	(95% CI)
**Denmark**^**c**^	320,846	29.4 [4.8]	0.37	(0.35–0.40)	0.60	(0.57–0.62)	0.41	(0.39–0.43)	1.22	(1.18–1.25)	0.71	(0.68–0.74)	0.43	(0.41–0.46)
**Norway**^**d**^	301,833	30.0 [4.9]	0.45	(0.43–0.47)	0.88	(0.85–0.91)	0.53	(0.50–0.55)	0.75	(0.71–0.87)	0.39	(0.36–0.41)	0.30	(0.28–0.32)
**Italy-Emilia Romagna**^**e**^	46,445	29.7 [5.1]	0.31	(0.25–0.36)	1.03	(0.93–1.12)	0.39	(0.33–0.45)	0.51	(0.45–0.58)	0.30	(0.25–0.35)	0.39	(0.33–0.45)
**Tuscany**	157,916	32.3 [5.0]	0.27	(0.25–0.30)	2.22	(2.15–2.29)	0.31	(0.29–0.34)	0.63	(0.59–0.66)	0.40	(0.37–0.43)	0.36	(0.33–0.39)
**The Netherlands**	14,607	31.8 [4.9]	0.38	(0.28–0.48)	1.10	(0.93–1.27)	0.41	(0.31–0.51)	0.29	(0.21–0.38)	0.14	(0.08–0.20)	0.14	(0.08–0.20)
**United Kingdom**^f^	182,920	30.1 [6.0]	0.44	(0.41–0.47)	1.01	(0.96–1.05)	0.45	(0.42–0.48)	0.75	(0.72–0.79)	0.61	(0.57–0.64)	0.36	(0.33–0.39)
**Wales**	58,106	27.6 [6.1]	0.37	(0.32–0.42)	0.71	(0.64–0.78)	0.43	(0.37–0.48)	0.97	(0.89–1.05)	0.69	(0.62–0.76)	0.44	(0.39–0.49)
**Total**	**1,082,673**													

^a^ at the start of pregnancy

^b^ standard deviation

^c^ 1 Jan 2004–31 Dec 2009

^d^ 1 Jan 2005–31 Dec 2010

^e^ 1 Jan 2008–31 Dec 2010

^f^ excluding Wales

**Fig 1:**
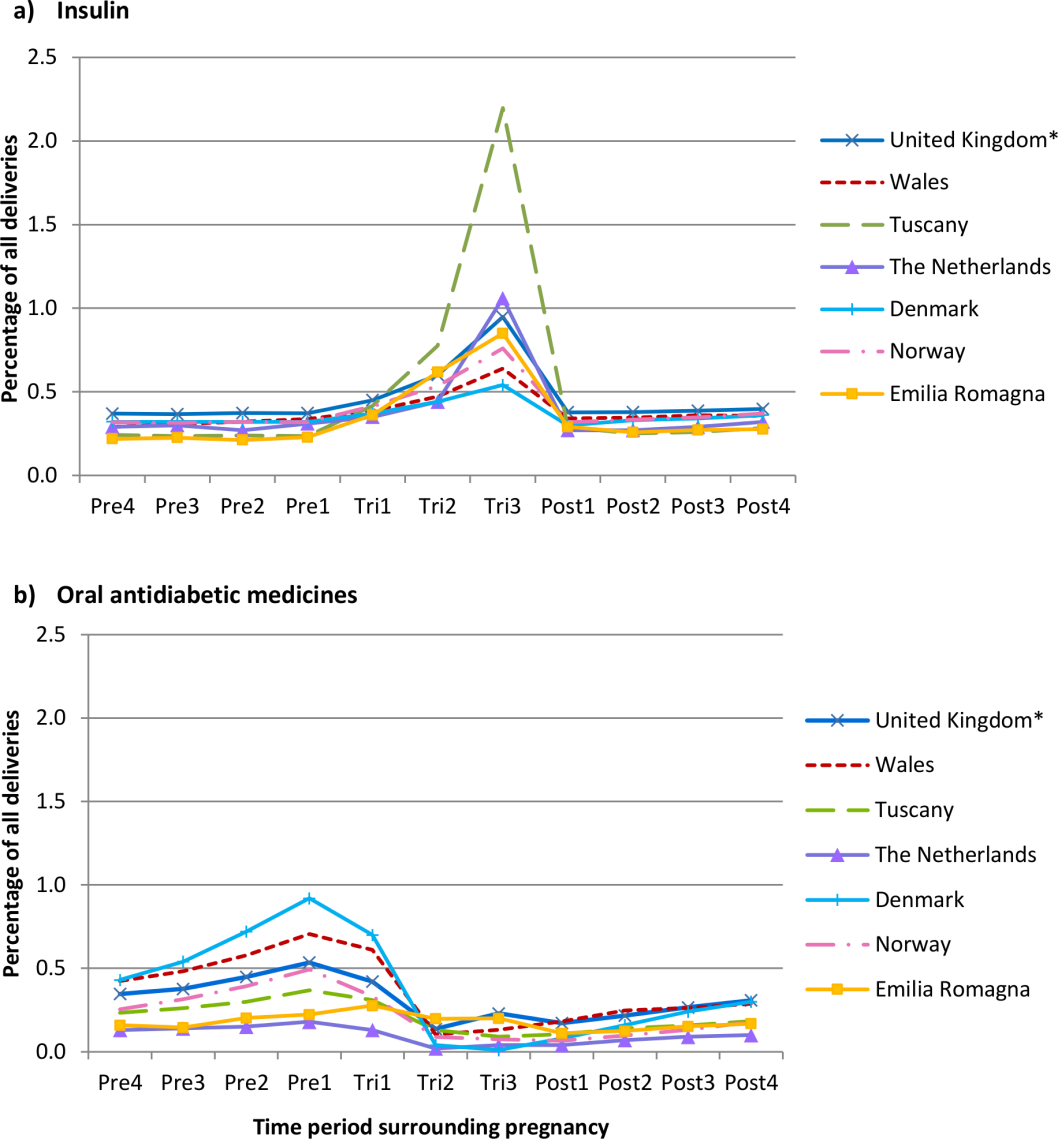
Prevalence of a) insulin and b) oral antidiabetic medicine prescribing in women with a delivery, between 2004 and 2010, during each of the 3-month time periods.

The prevalence of pregestational insulin prescribing during the year before pregnancy varied and was lowest in Tuscany (0.27% CI_95_ 0.25–0.30) and highest in Norway (0.45% CI_95_ 0.43–0.47) (Table 1). During pregnancy, insulin prescribing increased in all regions (Table 1), ranging from a 62% increase in Denmark to a 722% increase in Tuscany, which was higher than all other regions and took the prevalence of insulin prescribing to 2.2% of all deliveries during the third trimester (Fig 1A). In most regions, during the year following pregnancy, the prescribing of insulin declined to levels similar to those pre-pregnancy. Age-standardisation of the figures for insulin had little impact on the prevalence of prescribing for the majority of regions but did reduce the prevalence during the third trimester in Tuscany from 2.2% to 1.7% (S1 Fig).

Prescribing of oral antidiabetic medicines in all regions was most common during the year before pregnancy (Table 1). Regional variation, however, was observed in the prevalence of prescribing during this time and prescribing was highest in Denmark (1.22% CI_95_ 1.18–1.25) and lowest in the Netherlands (0.29% CI_95_ 0.21–0.38). The level of oral antidiabetic medicine prescribing declined during pregnancy and was lowest during the second and third trimesters (Fig 1B). Post pregnancy, prescribing increased slightly but remained considerably lower than the pre-pregnancy levels.

**Fig 2:**
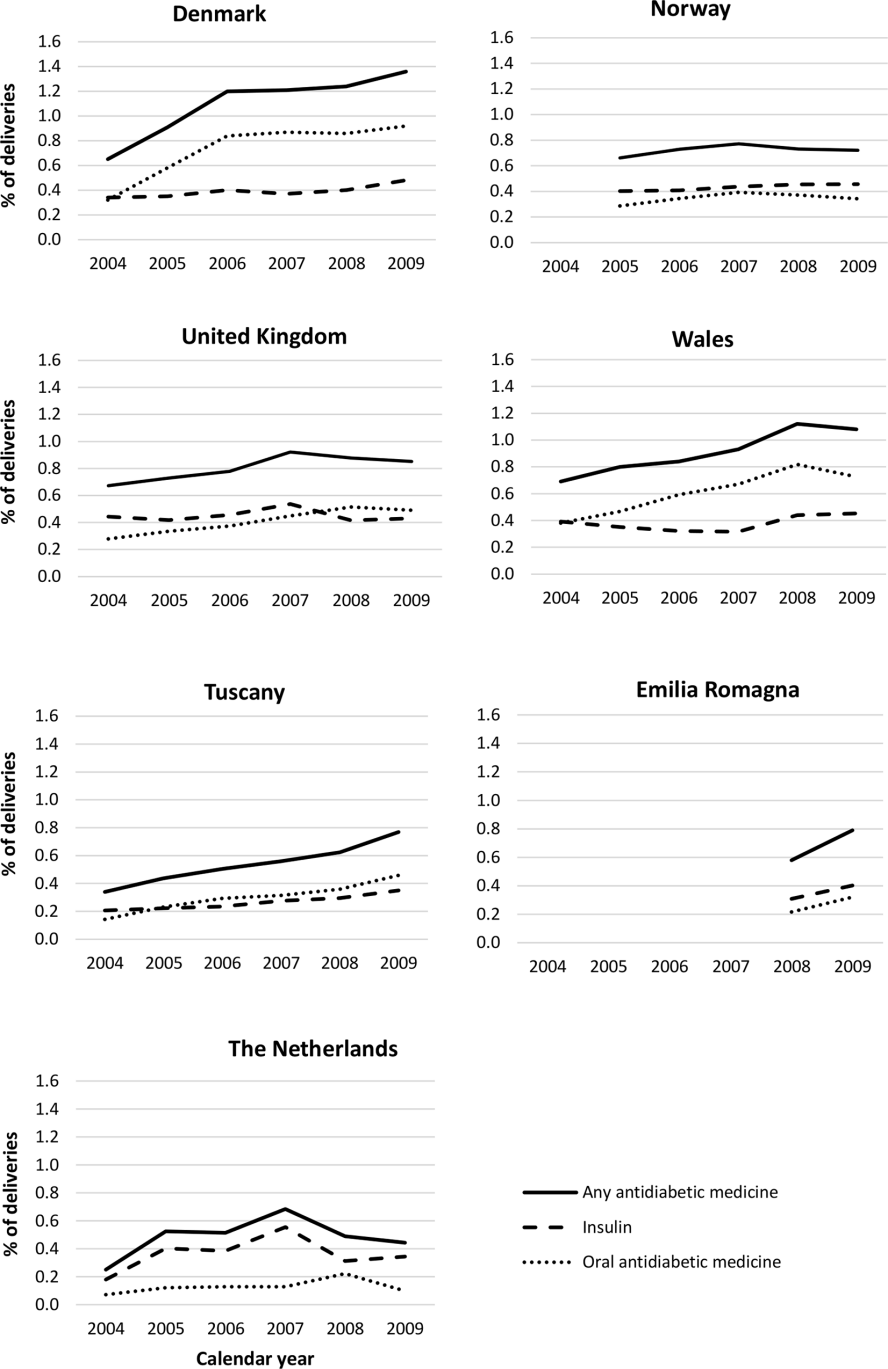
Trends in the prescribing of antidiabetic medicines during the first trimester of pregnancy by region and calendar year.

During the study period, increases were observed in some regions in the percentage of deliveries where the woman received a prescription for an antidiabetic medicine during the first trimester of pregnancy (Fig 2). Similar trends were also observed during the year before the start of pregnancy (S2 Table). In general, the larger increases were seen in the prescribing of oral antidiabetic medicines, particularly metformin, although some increases were observed in the prescribing of insulins.

#### Insulin prescribing

**Fig 3:**
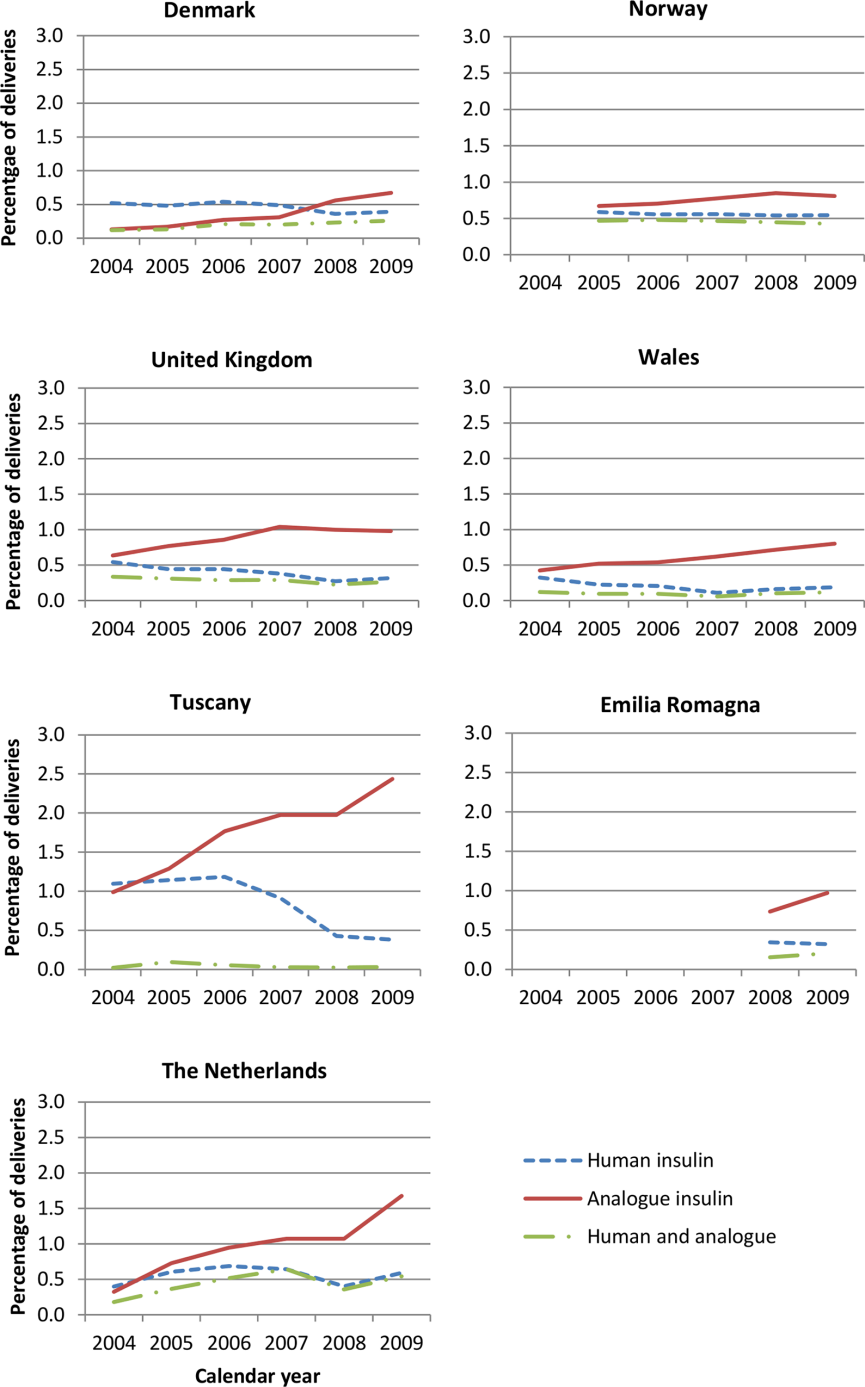
Trends in the prescribing of insulins during pregnancy by region and calendar year.

**Fig 4:**
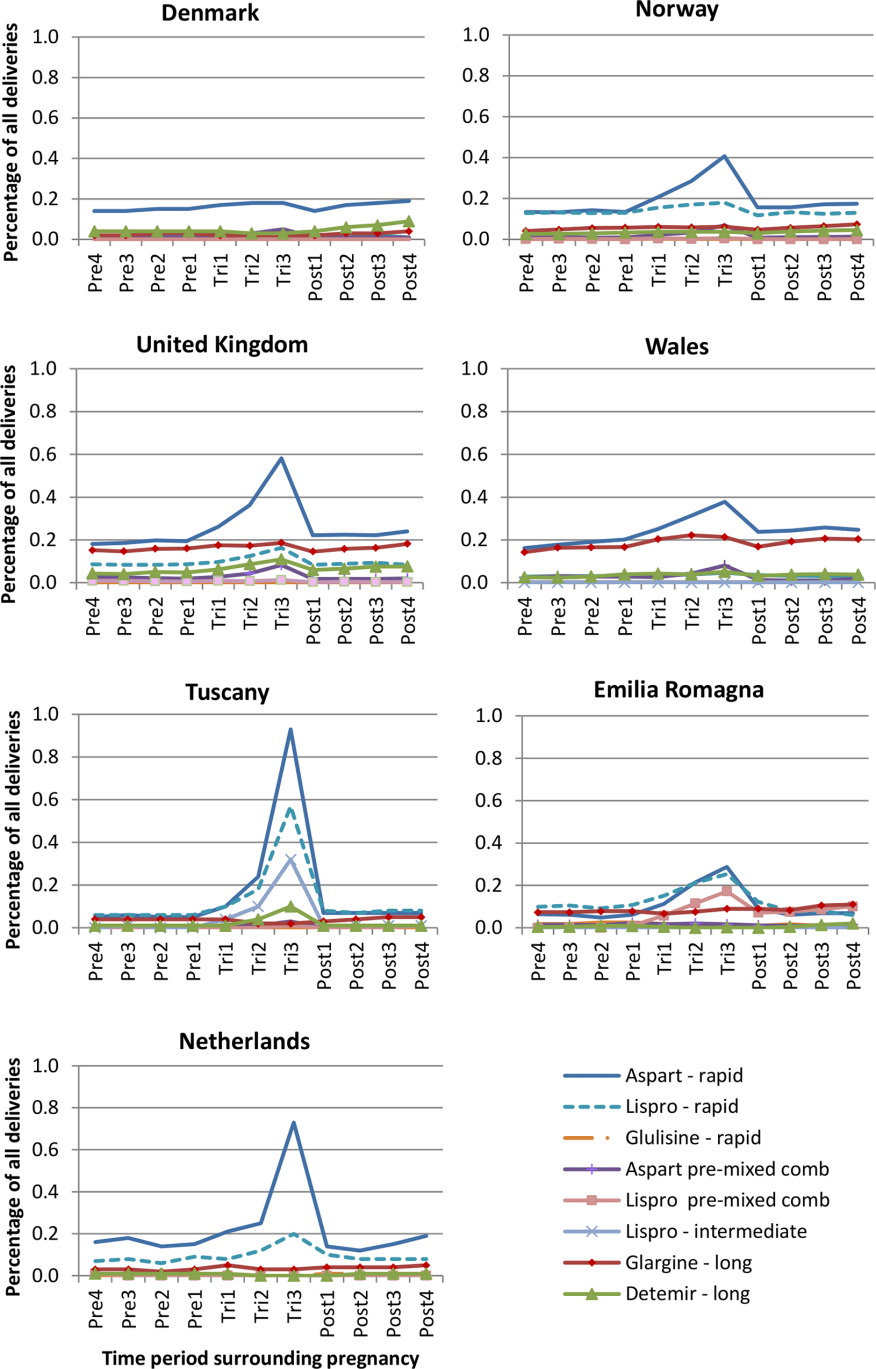
Breakdown of the type of analogue insulin prescribed during each of the time periods in each region.

During the study period, the prescribing of insulin analogues increased and a decline was observed in the prescribing of human insulins. Analogue insulins were the insulins most commonly prescribed during all 3-month time periods from 2005, with the exception of Denmark where until 2008 human insulins were the most popular (Fig 3). Of those prescribed an insulin during pregnancy, between 50.5% (Denmark) and 88.8% (Netherlands) received an insulin analogue alone or in combination with human insulin. In all regions, rapid-acting insulin aspart was the most commonly prescribed analogue insulin (Fig 4). In all regions, analogue insulins made up the majority of prescriptions for a long-acting insulin, with only a small number of long-acting human insulins prescribed/dispensed. Insulin detemir was the most frequently dispensed long-acting analogue used in Denmark, whilst in other regions it was insulin glargine. The percentage of women receiving a prescription during pregnancy for both analogue and human insulin ranged from ~10% in Wales to 40% in Norway (data not shown). In some regions, a small increase in the percentage of women receiving both analogue and human insulins was observed during pregnancy, and particularly the first trimester.

#### Oral antidiabetic medicine prescribing

During the study period approximately 98% of all oral antidiabetic prescriptions in Denmark, Norway, Wales and the rest of the UK were for metformin and there was little variation in this percentage during the time periods before, during and after pregnancy. In the Netherlands, the overall percentage was closer to 95%, although 100% of oral antidiabetic prescriptions dispensed during the second and third trimesters were for metformin. In the Italian regions, prescribing practices differed, with metformin making up around 90% of all oral products during the year before pregnancy, this then declined during pregnancy to approximately 75% during the third trimester and the reduction in metformin dispensing resulted in gliclazide and glimepinide subsequently making up a greater percentage of oral antidiabetic prescriptions. The Italian regions were the only ones to prescribe metformin in a pre-mixed combination with sulfonamides (ATC A10BD02) and these prescriptions made up 5% to 8% of metformin prescriptions during the year before pregnancy, increasing to ~15% during the third trimester.

### Pharmacologically treated gestational diabetes

**Fig 5:**
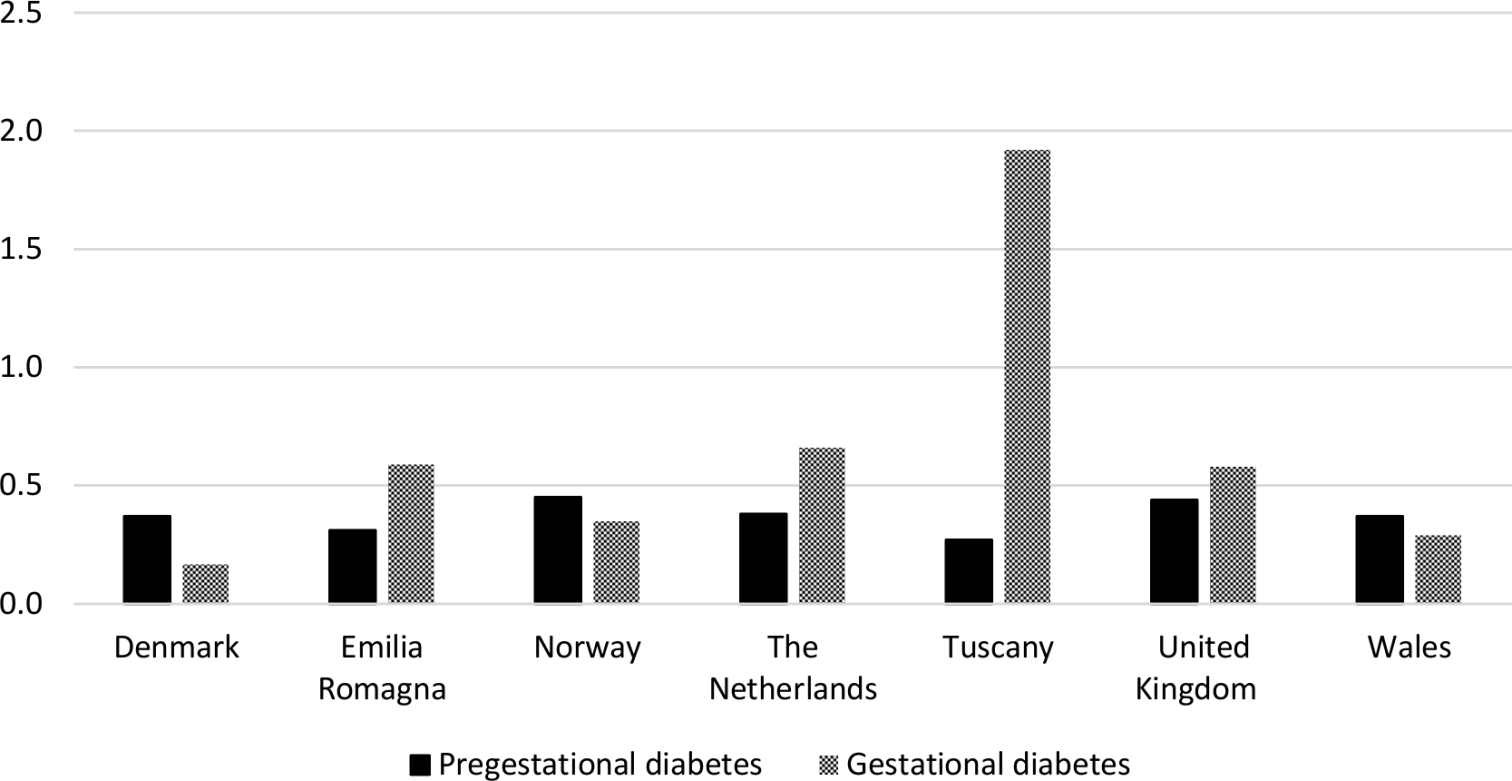
Percentage of deliveries where the woman received her first prescription for an antidiabetic medicine during the second or third trimester of pregnancy (as an indication of pharmacologically treated gestational diabetes) by region.

The percentage of deliveries where the mother received her first prescription for an antidiabetic medicine during the second or third trimester of pregnancy, indicative of pharmacologically treated gestational diabetes, ranged from 0.17% (CI_95_ 0.16–0.18) in Denmark to 1.92% (CI_95_ 1.85–1.99) in Tuscany (Fig 5).

**Fig 6:**
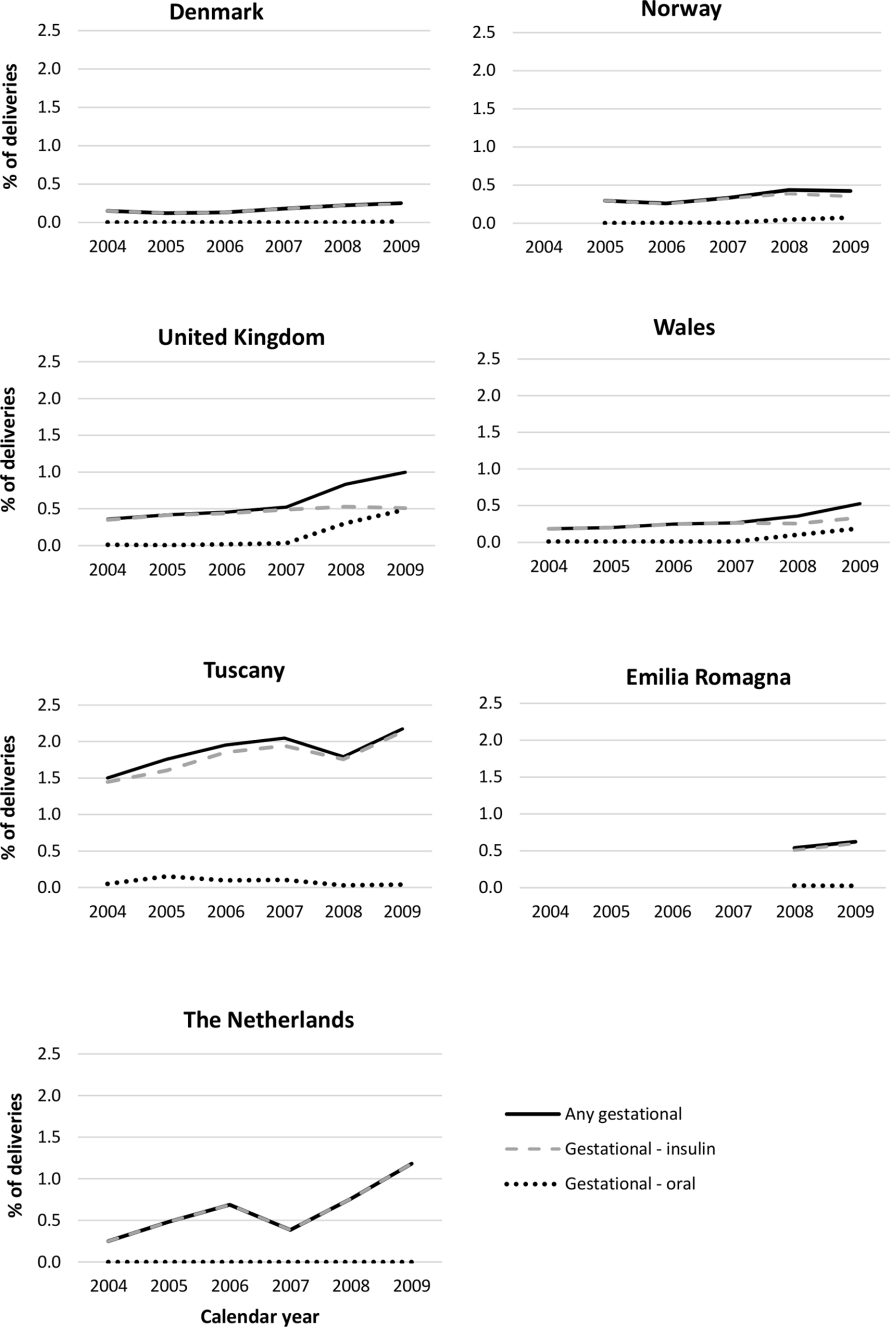
Percentage of pregnancies where the woman received her first prescription for an antidiabetic medicine during the second or third trimester of pregnancy (as an indication of pharmacologically treated gestational diabetes) by region and calendar year.

During the study period, increases were observed in the percentage of deliveries where the woman received her first prescription for an antidiabetic medicine during the second/third trimesters of pregnancy (Fig 6). Changes were also observed, in some regions, in the choice of treatment for gestational diabetes. Between 2004 and 2007, in all regions (excluding Emilia Romagna where data were not available), over 90% of women who received their first prescription for an antidiabetic medicine during the second or third trimester, received insulin. In Norway, Wales and the rest of the UK this declined in 2008 and by 2009 it had reduced to 83%, 64% and 50% respectively, with oral antidiabetic medicines being much more commonly prescribed. In all other regions no increase in the prescribing of oral products was observed up to 2010. In all regions, the prevalence of pharmacologically treated gestational diabetes increased with higher maternal age at the start of pregnancy (S2 Fig). In Italy, the Netherlands and the UK, a larger percentage of women with a delivery receiving prescriptions for an antidiabetic medicine received them for the treatment of gestational diabetes than for the treatment of pregestational diabetes (Fig 5), whilst in other regions pregestational diabetes made up a larger percentage of those receiving prescriptions for an antidiabetic medicine.

## Discussion

This study identified regional variations within Europe in the prevalence of prescribing for pregestational diabetes. During the seven year study period, increases were observed in some European regions in the prevalence of prescribing of antidiabetic medicines during the year before pregnancy and during the first pregnancy trimester. As insulin is only prescribed for the treatment of diabetes, the majority of regional variation will reflect differences in the prevalence of mainly type 1 but also type 2 diabetes and differences in the severity of type 2 diabetes. In addition, it is likely that there will be some heterogeneity between regions in whether women with type 2 diabetes are treated pharmacologically. The prevalence of insulin prescribing observed in this study during the year before pregnancy in Norway, was in line with the prevalence of pregestational type 1 diabetes reported in a study using data from the Medical Birth Register of Norway.[3] In Wales, the prevalence of insulin prescribing during the year before pregnancy was in line with the prevalence of pregestational type 1 and type 2 diabetes in a study reporting on England, Wales and Northern Ireland by Macintosh *et al*.[11] The prevalence of prescribing for the rest of the UK in our study, however, was found to be higher than in the Macintosh study which may in part be explained by our study covering a later time period (2004–2010 compared with 2002–2003). To our knowledge no studies have reported on the prevalence of pregestational diabetes in Denmark, the Netherlands or Italy.

Insulin analogues were the most commonly prescribed and their use increased during the study period in line with a reduction in human insulin prescribing. In all regions, fast-acting insulins were the insulins most commonly prescribed. Rapid-acting insulin analogues had the highest prescribing prevalence in all regions, with rapid-acting insulin aspart and lispro being the most frequently prescribed. Little is still known about the safety of insulin analogues when used in pregnancy, however both these products have a Food and Drug Administration (FDA) pregnancy category B. Evidence from a large trial for insulin aspart and retrospective and observational studies for insulin lispro have suggested that they are equivalent to human insulin in terms of fetal outcomes[9] but further data are needed. In addition, rapid-acting insulin analogues act faster than human insulins. Insulin glargine was the second most commonly prescribed insulin analogue in the two UK databases and was less frequently prescribed in the other regions. In the UK, the level of insulin glargine prescribing remained constant during pregnancy, suggesting that its use was restricted to the treatment of pre-existing diabetes rather than gestational diabetes. During the study period, however, the 2008 UK NICE guidelines advised that there was insufficient evidence about the use of analogue insulin glargine during pregnancy and recommended that its use should be avoided until more data were available on its safety.[12] In all regions, analogue insulins made up the majority of prescriptions issued/dispensed for a long-acting insulin. This is consistent with the fact that analogue long-acting insulins have been shown to improve HbA1c and decrease the risk of nocturnal and severe hypoglycaemia compared to human insulins.[32]

Regional variations were observed in the prevalence of pharmacologically treated gestational diabetes. During the seven year study period, increases were observed in some European regions in the prevalence of prescribing for gestational diabetes during the second and third trimesters of pregnancy.These regional variations will reflect differences in the prevalence of the disease with different ethnic populations, different inclusion criteria for screening programmes (S3 Table), as well as differences in clinicians’ perception of the significance of gestational diabetes and its treatment.[13, 33] The higher prevalence of gestational diabetes observed in Tuscany, compared with the more northern regions of Europe, was in line with the trend reported in a review article by Buckley *et al*. of a higher prevalence of gestational diabetes in southern Europe compared with northern Europe,[13] but this may also reflect the fact that during the study period all women were offered screening in Tuscany and not just those considered to be at high risk. Age-standardisation also demonstrated that maternal age explained some of the regional differences observed. Despite the higher prevalence of antidiabetic medicine prescribing during the later stages of pregnancy in Tuscany, the levels post-pregnancy were similar to those before pregnancy, indicating that few undiagnosed pregestational cases of diabetes were diagnosed during pregnancy. During the study period, the World Health Organization recommended that the majority of cases of gestational diabetes should be treated by diet alone and that insulin therapy should be used when medical nutrition therapy failed to maintain adequate glucose levels.[34] The fact that our study identified women who were pharmacologically treated and not women treated by diet alone, will in part explain why the prevalence of gestational diabetes in our study was, for the majority of regions, lower than those reported in the Buckley review, which were in the range of between 2% and 6% of all pregnancies[13] compared with 0.2% to 1.9% in our study.

Within-country regional variation in the prevalence of pharmacologically treated gestational diabetes was also observed in both Italy and the United Kingdom. In Italy, variations were likely to be the result of different screening policies in the two regions during the study period (the Tuscany policy at that time was to screen all women whereas in Emilia Romagna only high risk women were offered screening–S2 Table). The lower prevalence of pharmacologically treated gestational diabetes observed in Wales, compared with the rest of the UK, could in part result from differences in the implementation of the screening guidelines and whether pharmacological treatment was offered, in addition to population demographics, with Wales having a lower percentage of individuals from those ethnic backgrounds considered to be at a higher risk of diabetes[35] and a younger mean age at delivery.

Oral antidiabetic medicines were found to largely consist of metformin and were most commonly prescribed during the year before pregnancy and the first trimester, with low levels of prescribing during the second and third trimesters of pregnancy. It is likely that during the year before pregnancy some of this prescribing will have been for the treatment of conditions other than diabetes, such as infertility and PCOS. In Norway, Wales and the rest of the UK, increases were observed in the prescribing of metformin for the treatment of gestational diabetes towards the end of the study period, from 2008 onwards. This coincided with the introduction of the NICE guidelines in 2008 in the UK and the publication of a randomised trial looking at perinatal outcomes comparing metformin and insulin, which concluded that metformin, with or without insulin, is a safe and effective treatment for women with gestational diabetes who meet the standard criteria for starting insulin therapy.[36] The results of this trial were also referenced in one of the Norwegian guidelines and may explain why Norway also saw an increase in oral antidiabetic prescribing for the treatment of gestational diabetes from 2008.

Future work is needed to explain the wide range of metformin prescribing patterns observed during the year before the start of pregnancy and to investigate the indications for which metformin is being prescribed. A study by Lawrence *et al*., evaluating data from Health Maintenance Organizations in the United States, found only 13% of women receiving a prescription for metformin in the 120 days before pregnancy had a diagnosis of diabetes and 67% had a diagnosis of polycystic ovaries or female infertility.[37] It is not known whether the same would be true for the European data, but this may explain the high prescribing rates observed in our study prior to pregnancy and continuing into the first trimester. The prescriptions we identified during the first trimester may in some cases have been issued/dispensed before the pregnancy was recognised. The high level of discontinuation of metformin during pregnancy in our study is in line with the US study, where two-thirds of women did not go on to use any antidiabetic medicines during pregnancy.

This study used a common protocol and captured over 21,500 deliveries between 2004 and 2010 where the woman received a prescription for an antidiabetic medicine during pregnancy or during the one year either side of pregnancy. Prescription information was recorded independently by the prescriber or dispensing pharmacist, preventing maternal recall bias. No data, however, were available on whether the woman actually took the medicine, the precise timing at which she took it and whether she took it as instructed. In the databases covering Wales and the rest of the UK, all prescriptions issued in primary care were captured, whereas in other regions it was only prescriptions actually dispensed. It is possible that some women who are issued prescriptions may not get them dispensed; this is less likely for insulins for type 1 diabetes than for some oral products such as metformin.

This study did not look at prescription duration or take into account the fact that prescriptions issued/dispensed during one 3-month time period could have been continued during the following 3-month time period and this may have resulted in an underestimation of exposure during some time periods. The extent of misclassification resulting from this will partly be dependent on the quantity and duration of time covered by a single prescription is issued in each of the regions. In around 15% of deliveries, part of the 1-year time period following the first pregnancy overlapped with part of the 1-year time period prior to the start of a subsequent pregnancy. This will not have influenced the prescribing during the actual pregnancy but for the deliveries where this occurred, exposure during part of the year following the first delivery may have been influenced by the fact the woman had become pregnant again. Using the data available within the databases, it was not possible to distinguish between women being treated for type 1 and type 2 diabetes, especially as women with type 2 diabetes are increasingly being treated with insulin. It was also not possible to identify women who were being treated for diabetes by changes to diet alone, so the figures reported here do not represent the full extent to which pregnant women are affected by diabetes.

A multicentre prospective study in Europe has predicted that the prevalence of type 1 diabetes in children aged under 15 years will increase by 70% between 2005 and 2020.[38] This combined with the emergence of type 2 diabetes in children and younger women of childbearing age will result in further increases in the prevalence of pregestational diabetes in the future. It will become increasingly necessary to reinforce public health efforts to prevent diabetes through exercise and weight control, to make sure that the most effective treatments in terms of type of antidiabetic medicine are being prescribed, that women are offered and access preconception care to optimise glycaemic control for pregestational diabetes, and that effective screening for gestational diabetes leads to its optimal treatment for all affected women.

## Supporting Information

S1 Fig Prevalence of insulin prescribing in women with a delivery, between 2004 and 2010, during each of the 3-month time periods after age-standardisation. (PDF)

S2 Fig Trends in the prescribing of insulins during pregnancy by region and calendar year. (PDF)

S1 File Underlying numerical values behind the figures, tables and graphs reported in the manuscript. (XLSX)

S1 Table Overview of the databases accessed for the study. (DOCX)

S2 Table Changes in the prevalence of prescribing of antidiabetic medicines between 2004 and 2009 in the year before pregnancy and during the first trimester of pregnancy in the different databases. (DOCX)

S3 Table Summary of the guidelines for screening for gestational diabetes in each region. (DOCX)
