# Supplementary figures and images for: Prescribing of Antidiabetic Medicines before, during and after Pregnancy: A Study in Seven European Regions
Source: PLoS One. 2016 May 18;11(5):e0155737. doi: 10.1371/journal.pone.0155737 (PMC4871589; doi:10.1371/journal.pone.0155737)

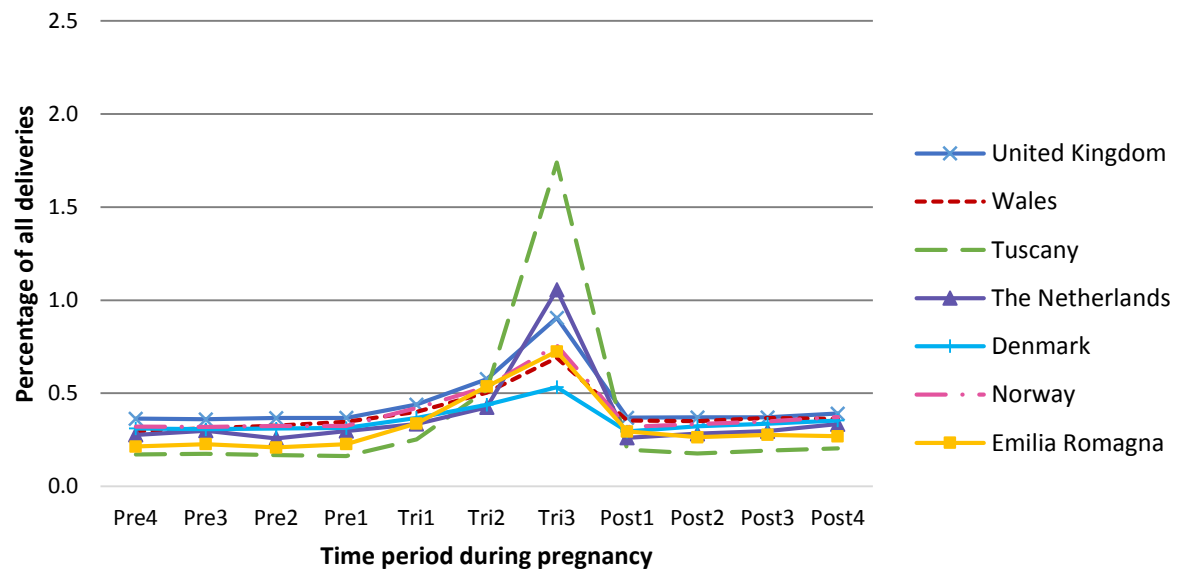

Supplement: S1 Fig — (PDF) [file pone.0155737.s001.pdf]

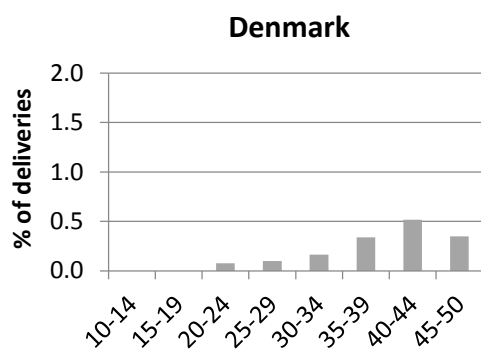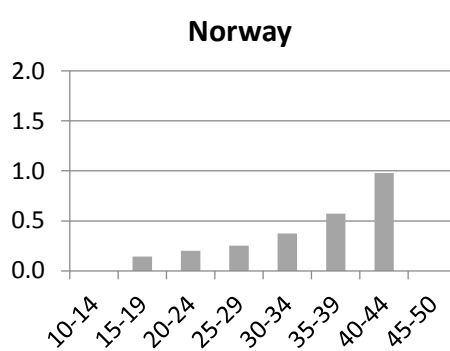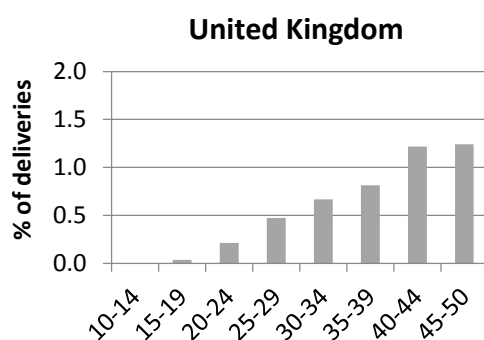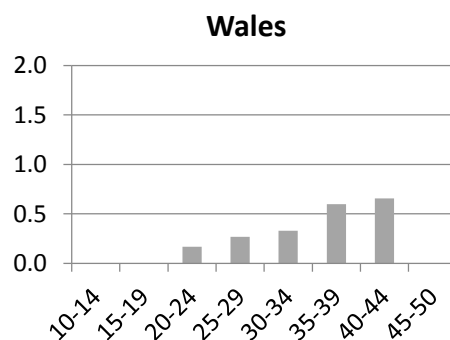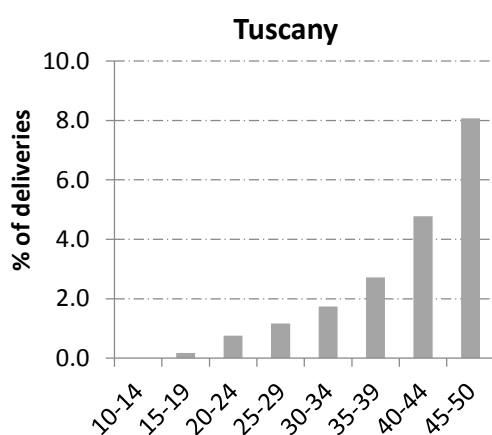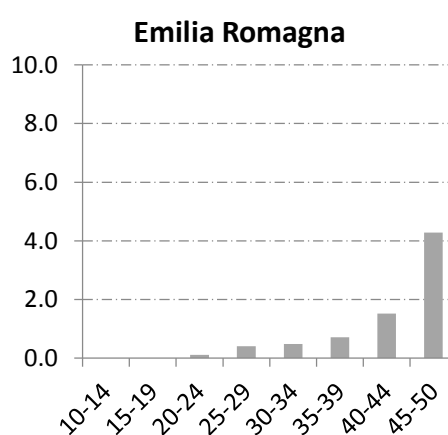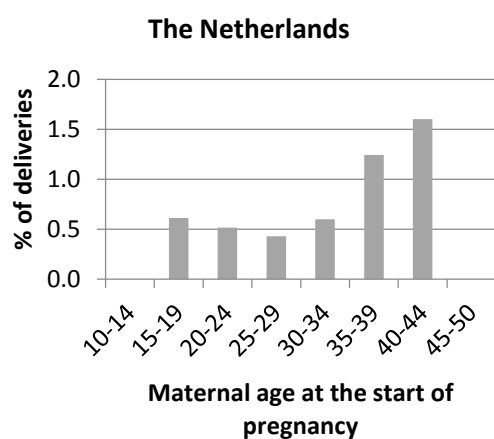

Maternal age at the start of pregnancy

Supplement: S2 Fig — (PDF) [file pone.0155737.s002.pdf]
