# Supplementary material for: Prescribing of Antidiabetic Medicines before, during and after Pregnancy: A Study in Seven European Regions
Source: PLoS One. 2016 May 18;11(5):e0155737. doi: 10.1371/journal.pone.0155737 (PMC4871589; doi:10.1371/journal.pone.0155737)
Supplement: S1 Table — (DOCX) [file pone.0155737.s004.docx]

| **Country/Region** | **Netherlands** | **Denmark** | **Norway** | **Italy -**  **Emilia Romagna** | **Italy -**  **Tuscany** | **United Kingdom^a^** | **Wales** |
| --- | --- | --- | --- | --- | --- | --- | --- |
| **Involves database linkage** | No | Yes | Yes | Yes | Yes | No | Yes^b^ |
| **Coverage** | Regional | National | National | Regional | Regional | ~8.5% of UK population | ~40% of GP practices in Wales |
| **Population base** | 500,000 | ~5,000,000 | ~4,800,000 | 4,200,000 | 3,700,000 | ~5,000,000^c^ | 2,000,000 |
| **Database for live & stillbirth pregnancy identification** | IADB.nl  database | Danish National  Birth Registry | Medical  Birth Registry  of Norway | Certificate of Delivery Assistance (CeDAP) | Certificate of Delivery Assistance Hospital Discharges Registry | Clinical Practice Research Datalink  (CPRD)^d^ | National Community Child Health Database  (NCCHD) |
| **Database for medicine use data** | IADB.nl  database | Danish  Prescription Registry | Norwegian Prescription Database | Emilia-Romagna Prescription Database (ERPD) | Tuscany Prescription Database  (TPD) | Clinical Practice Research Datalink | The General Practice (GP) Dataset |
| **Source for medicine use data** | Pharmacy dispensing | Pharmacy dispensing | Pharmacy  dispensing | Pharmacy  Dispensing^e^ | Pharmacy dispensing and Healthcare Facilities Dispensing^e^ | GP practice prescribing | GP practice prescribing |
| **Capture GP prescribing** | Yes | Yes | Yes | Yes | Yes | Yes | Yes |
| **Capture hospital inpatient prescribing** | No | No | No | No | No | Some | Some |
| **Date of last menstrual period recorded** | Estimated  for all | Calculated from gestational age | Yes^f^ | Calculated from gestational age | Calculated from gestational age | Yes for 40%  Estimated for 60% | Yes for 80%  Estimated for 20% |

^a^ Excluding practices in Wales to avoid duplication of pregnancies in the database contributing data for Wales; ^b^ Secure Anonymised Information Linkage (SAIL) databank; ^c^ The size of the population captured by the CPRD has grown steadily over time and was approximately 5.0 million in May 2012; ^d^ Previously the General Practice Research Database (GPRD); ^e^ Only products reimbursed by the Italian National Health Service; ^f^ For this study ultrasound estimations were used to determine gestational age and start of the pregnancy
