# Supplementary material for: Prescribing of Antidiabetic Medicines before, during and after Pregnancy: A Study in Seven European Regions
Source: PLoS One. 2016 May 18;11(5):e0155737. doi: 10.1371/journal.pone.0155737 (PMC4871589; doi:10.1371/journal.pone.0155737)
Supplement: S2 Table — (DOCX) [file pone.0155737.s005.docx]

| **Country/region** | **Any antidiabetic medicine during year before pregnancy** | | | | **Any antidiabetic medicine during the first trimester of pregnancy** | | | |
| --- | --- | --- | --- | --- | --- | --- | --- | --- |
|  | **2004** | | **2009** | | **2004** | | **2009** | |
| **Denmark** | 0.86 | (0.78-0.93) | 2.14 | (1.89-2.38) | 0.65 | (0.58-0.71) | 1.36 | (1.16-1.55) |
| **Norway** | NA | NA | 1.12 | (1.04-1.20) | NA | NA | 0.72 | (0.65-0.79) |
| **Italy – Emilia Romagna** | NA | NA | 0.60 | (0.49-0.72) | NA | NA | 0.79 | (0.66-0.92) |
| **Italy – Tuscany** | 0.50 | (0.41-0.59) | 1.12 | (0.99-1.25) | 0.34 | (0.27-0.41) | 0.77 | (0.66-0.88) |
| **The Netherlands** | 0.39 | (0.16-0.63) | 0.79 | (0.40-1.17) | 0.25 | (0.07-0.44) | 0.44 | (0.15-0.73) |
| **United Kingdom** | 0.86 | (0.75-0.97) | 1.18 | (1.06-1.30) | 0.67 | (0.58-0.77) | 0.85 | (0.75-0.96) |
| **Wales** | 0.88 | (0.69-1.07) | 1.50 | (1.26-1.75) | 0.69 | (0.53-0.86) | 1.08 | (0.88-1.29) |
