# Supplementary material for: Prescribing of Antidiabetic Medicines before, during and after Pregnancy: A Study in Seven European Regions
Source: PLoS One. 2016 May 18;11(5):e0155737. doi: 10.1371/journal.pone.0155737 (PMC4871589; doi:10.1371/journal.pone.0155737)
Supplement: S3 Table — (DOCX) [file pone.0155737.s006.docx]

| **Country/region and guideline** | **Summary of screening practice** |
| --- | --- |
| **Denmark**  Danish National Guidelines for pregnant women^[38]^ | Based on 7 risk factors:   - - Previous gestational diabetes   - Family members with type 1 or type 2 diabetes   - BMI of 27 or more   - Previous infant with birth weight at +4500 grams   - Multiple pregnancy   - Polycystic ovaries   - Glucosuria   Women with glucosuria at any time in Denmark have an oral glucose test performed immediately. Those with one risk factor are screened at 28-30 weeks and those with 2 or more risk factors at 18-20 weeks and 28-30 weeks. Overall about one third of pregnant women are screened. |
| **Norway**  Norsk gynekologisk Forening  Guidelines^[39]^ | Based on 3 risk categories:  **Group A**   - Previous gestational diabetes - First degree relatives with Diabetes type 1 or 2 - Immigrants, especially from N Africa or Indian subcontinent - Older than 35 years - Pre-pregnant BMI of 27 or more   For this group, the guidelines recommend screening with glucose tolerance test as early as possible in pregnancy, and repeat the test in week 28-30 if the first test was negative.  **Group B**   - Glucosuria, especially if this is repeated, and if there is no obvious explanation.   For this group, the guidelines recommend screening with glucose tolerance test when glucosuria is diagnosed. If the first test is negative, it should be repeated after 4-6 weeks (or sooner if she is in third trimester)  **Group C**   - Polyhydramnion and/or large increase in estimated intrauterine growth - Previous large baby (>4500 grams) - Random fasting blood glucose is between 6.1 and 7.0 mmol/l - Congenital anomalies in previous pregnancies - Previous intrauterine death   In this group, the guidelines recommend screening with glucose tolerance test as an option. |
| **United Kingdom**  **(including Wales)**  NICE clinical guideline (2008)^[12]^ These have been revised in February 2015^[17]^ | Based on 5 risk factors:   - Body mass index above 30 kg/m2 - Previous macrosomic baby weighing 4.5 kg or above - Previous gestational diabetes - First-degree relative with diabetes - Family origin with a high prevalence of diabetes:   - South Asian (specifically women whose country of family origin is India, Pakistan or Bangladesh)   - Black Caribbean   - Middle Eastern (specifically women whose country of family origin is Saudi Arabia, United Arab Emirates, Iraq, Jordan, Syria, Oman, Qatar, Kuwait, Lebanon or Egypt).   The above risk factors should be determined at the booking appointment and women with any of these risk factors should be offered testing for gestational diabetes. |
| **Tuscany**  Carpenter and Coustan Criteria^[40]^ | During the study period, screening with oral glucose tolerance test (OGTT) 50g was offered to all women between 24 and 28 gestational weeks, regardless of risk factor status, free of charge. In case of women at high risk (Previous gestational diabetes; Body mass index above 30 kg/m2; Blood glucose level 100-125mg/dl prior to or at the beginning of pregnancy) the screening was performed at 14-18 weeks and if negative was repeated at 24-28 weeks.  Since January 2013, universal screening is no longer offered in Tuscany. OGTT 75g is offered at 24-28 weeks and 3 blood tests to women with: positive first degree familial risk, age >35 years, BMI >25, fetal macrosomy in previous pregnancy, origin from higher risk areas (e.g. India, Bangladesh, Arabia, Giordania, Syria, Egypt). For women with previous gestational diabetes, BMI >30, fasting blood glucose 100/125, the same OGTT 75 g is offered at 16-18 weeks, to repeat, if negative, at 24-28 weeks. |
| **Emilia Romagna**  SNLG-ISS National system of Guidelines^[14]^ | Based on risk factors: Test offered to women who at 16-18 weeks gestation have at least one of the following   - Previous gestational diabetes - Body mass index above 30 kg/m2 - Blood glucose level 100-125mg/dl prior to or at the beginning of pregnancy |
| **The Netherlands**  Diabetes en Zwangerschap  NVOG (June 2010)^[41]^ | During the 1st trimester of pregnancy - blood-glucose level is measured (not-fasting level) in all pregnant women. Most women (>95%) have a level < 6.1 mmol/l. Those with a level > 6.1 mmol/l have a fasting glucose test and this is repeated during future visits.  During the 3rd trimester of pregnancy - women get a glucose tolerance test based on the following risk factors:   - BMI above 30 - Earlier child >4500 gram - woman born in the middle east or South Asia - maternal diabetes in a former pregnancy - polycystic ovary syndrome (PCOS) |
